# Image encryption using binary polarization states of light beam

**DOI:** 10.1038/s41598-023-41251-w

**Published:** 2023-08-28

**Authors:** Allarakha Shikder, Naveen K. Nishchal

**Affiliations:** Department of Physics, Indian Institute of Technology Patna, Bihta, Patna, 801106 India

**Keywords:** Optics and photonics, Physics

## Abstract

Optical image/data encryption techniques are mostly based on the manipulation of spatial distributions of light's amplitude, phase, and polarization. Information encoding with phase involves complex interferometric set-up and polarization encoding requires Stoke’s parameter measurement. Hence, they create difficulties in optical implementation. Considering the practical limitations, in this study, we demonstrate a method of single-shot intensity recording-based color image encryption by encoding the information in binary polarization states. The proposed method does not require Stoke parameter calculation. As a proof-of-concept, we demonstrated the technique with coherent and partially coherent light sources. Use of partially coherent light overcomes the speckle problem and makes the system cost-effective, useful for practical applications.

## Introduction

Recently, free-space optical communication has drawn lots of interest because of its benefits of low cost and high capacity^1,2^. The next major concern with the optical communication is the security of the communicated information. Regarding this various cryptosystems has been reported^3–8^. The aim to develop a cryptosystem is to protect the information from unauthorized access during its public release^3,4^. Optical cryptosystems offer benefits of being inherently two-dimensional information encoding and processing with the speed of light^5^. In several studies, efforts have been made to enhance the capabilities of optical cryptosystems^4–9^. Among them, optical cryptosystem based on double random phase encoding (DRPE)^10–12^, holographic techniques^13,14^, fractional Fourier transform domain encoding schemes^15,16^, polarization-based techniques^17–22^, using quick response code^23,24^, gyrator transform domain^24,25^, and use of metasurfaces^26,27^ are very popular.

Intensity, phase, and polarization are the main parameters of a light beam based on which different optical cryptosystems have been designed^5^. Characteristics of those parameters play a vital role in designing a cryptosystem by allowing effective encoding and decoding. High security is possible using phase encoding techniques but they rely on accurate wavefront control and measurement^7,17^ and this process also experiences quality deterioration when used in practice^5^. They introduce wavefront discontinuities, which bring unwanted artefacts introduced by diffraction, which degrades the decoding quality^28^. In cryptosystems based on polarization encoding, a similar issue occurs^19,20^.

An image encoding scheme using petal-like lattices has been reported where the information of a single pixel is encoded into the light beam of a petal-like lattices^29^. Thus, for encoding a full image multiple times encoding into the light beam in series are required corresponding to each pixel. That requires much time for a single image encoding and creates difficulty for instant information processing. Recently, array of vortex beam-based encryption system has been reported^30^, where information of an image is encoded in terms of the topological charge values of the vortices of the array. This process needs a complicated interferometric set-up for identifying the topological charge values for correct decryption. An intensity recording-based encryption scheme based on a multilayer array of vortex beams has been reported^31^ but the scheme is limited for binary images only. Based on spatial nonlinear optics, nonlinear encryption technique using self-phase modulation of a photorefractive crystal has been reported which is resistant to known plaintext attacks^32,33^. Recently, Xiao et al*.* proposed a physically enhanced ghost encoding scheme using dynamically and physically generated scaling factors as keys^34^. Most recently, an encryption scheme encoding two-dimensional information carriers for secure free-space optical transmission through dynamic and turbulent media has been reported^35^.

Several methods have been reported regarding polarization-based image encoding exploiting the property of spatial light modulators (SLM)^18,36–39^. In these schemes, a binary image has been used where each pixel is individually encrypted using exclusive OR (XOR) operation. In this method, each key pixel independently decodes its corresponding plaintext pixel. As a result, plaintext may be tracked even with partially correct key information. To overcome this issue, optical XOR operation with a phase retrieval algorithm has been reported where data decryption is not possible with a partial correct key^24^. In this scheme, pixel-to-pixel XOR operation has been achieved with a pair of SLMs inside two crossed polarizers. The requirement of two SLMs makes the cryptosystem costly and implementation involves complexity. Perfect pixel-to-pixel matching is difficult to achieve, otherwise, it introduces errors in the decoded data. Recently, spatially variable polarization image encryption techniques using single SLM has been reported which make practical implementation simpler but numerous intensity measurements for Stoke’s polarimetry calculation are still necessary, create difficulties for instant application^20,40^. These techniques used single random dataset as the encryption key, thus there is a scope for further enhancement of security by using additional keys.

In this study, we demonstrate a single-shot intensity recording-based color image encoding scheme using a single SLM. The method works under the double random encryption keys framework. Most of the earlier reported techniques employed two SLMs under coherent light illumination. In this case, a single SLM has been used under partially coherent light source. This not only makes the scheme cost-effective but reduces the speckle noise also. Here, we encode the information into binary polarization states. Therefore, for decoding the information multiple intensity recordings for Stoke’s parameters measurement are not required. Also, noise tolerance is high. A single-shot intensity recording through an analyzer is enough for decoding. The scheme being non-interferometric offers ease in optical implementation. We demonstrate the scheme using both coherent and partially coherent light sources.

## Principle

### Convert color image to binary data

The phase, intensity, and polarization of a light beam are controlled by its wavefront. Thus, it is easy to encode information in wavefront by modulating with a phase-only function (POF) containing values within − π to π. Here, a phase retrieval technique referred to as modified Gerchberg-Saxton algorithm (MGSA) is used to analyze a POF for encoding^30^. If *A*(*x*,*y*) denotes an input image corresponding to the* x*–*y* transverse coordinate then the POF, *Φ*(*x*,*y*) is expressed as,

1 $$ A(x,y) = \left| {FT\left\{ {\exp \left( {i\Phi } \right)} \right\}} \right|^{2} $$

where FT stands for Fourier transform operation. Data encoding is possible directly by modulating the wavefront with the POF using a phase-modulator device^6,20,21,41^. In case of the practical implementation of this process, it may reduce the quality of decoded data due to speckle noise^4,5^. This issue can be avoided by encoding the information of the POF into the light beam in terms of binary data. Here, each pixel value of the POF, *Φ*(*x*,*y*) is mapped to an array of integer number between -*R* to + *R*, according to the linear relation,

2 $$ X_{m,n} (x,y) = Int\left\{ {R\Phi (x,y)/\pi } \right\} $$

where the function “*Int*” stands for the nearest integer function, which returns the nearest integer number corresponding to any fractional value and *R* is a threshold value of those integer numbers. Thus, each pixel value of a POF of *p* × *q* pixels is converted into an array of *p* × *q* integer numbers having values between − *R* to + *R*. Next, a particular integer number *X*_m,n_(*x*,*y*) is converted to its equivalent binary values. Thus, the final achieved data is nothing but a combination of an array of numbers 0 and 1. It is possible to encode this binary data into the light beam in the form of binary polarization states where the encoded light beam returns low intensity corresponding to 0 and high intensity corresponding to 1 after propagating through an analyzer at a particular angle. The value of *R* should be chosen properly while taking experimental viability into account and making sure no significant information is lost.

### Generation of binary polarization states

**Figure 1:**
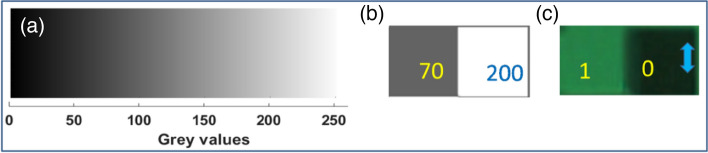
(**a**) Gray value from 0 to 255, (**b**) gray scale pattern for encoding binary polarization states, and (**c**) experimentally obtained intensity distribution corresponding to binary polarization states.

**Figure 2:**
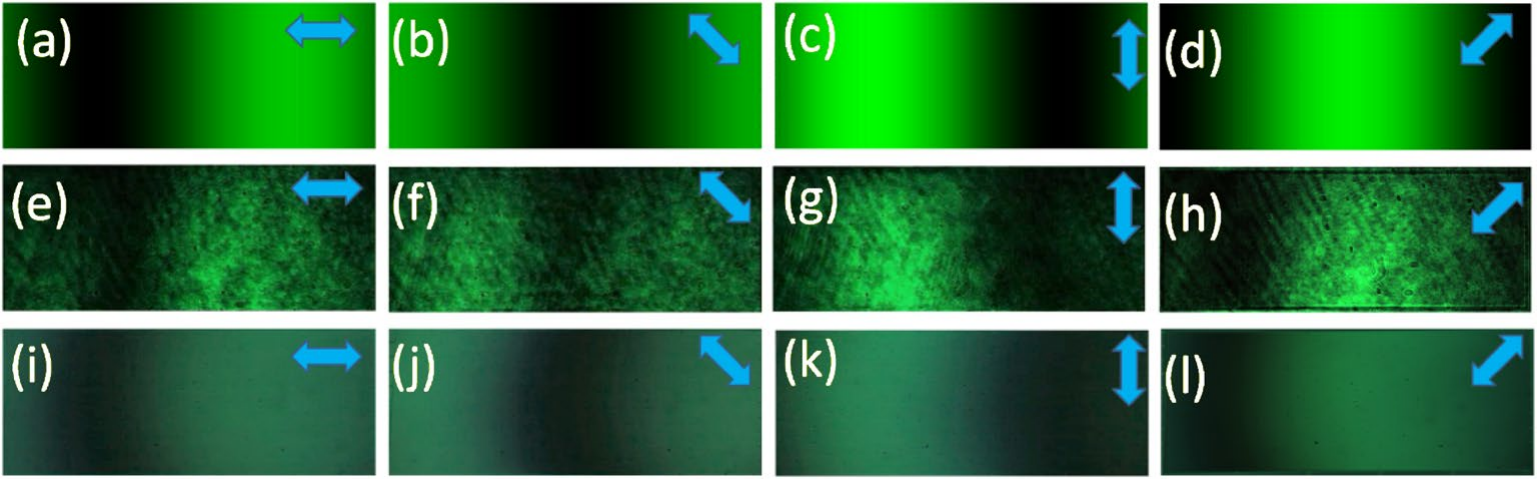
(**a**–**d**) Simulation results of intensity distributions for analyzer angles 0°, 45°, 90°, and 135°, respectively, (**e**–**h**) experimentally obtained intensity distributions with a coherent laser source for analyzer angles 0°, 45°, 90°, and 135°, respectively, (**i**–**l**) experimentally obtained intensity distributions with a light emitting diode for analyzer angles 0°, 45°, 90°, and 135°, respectively.

**Figure 3:**
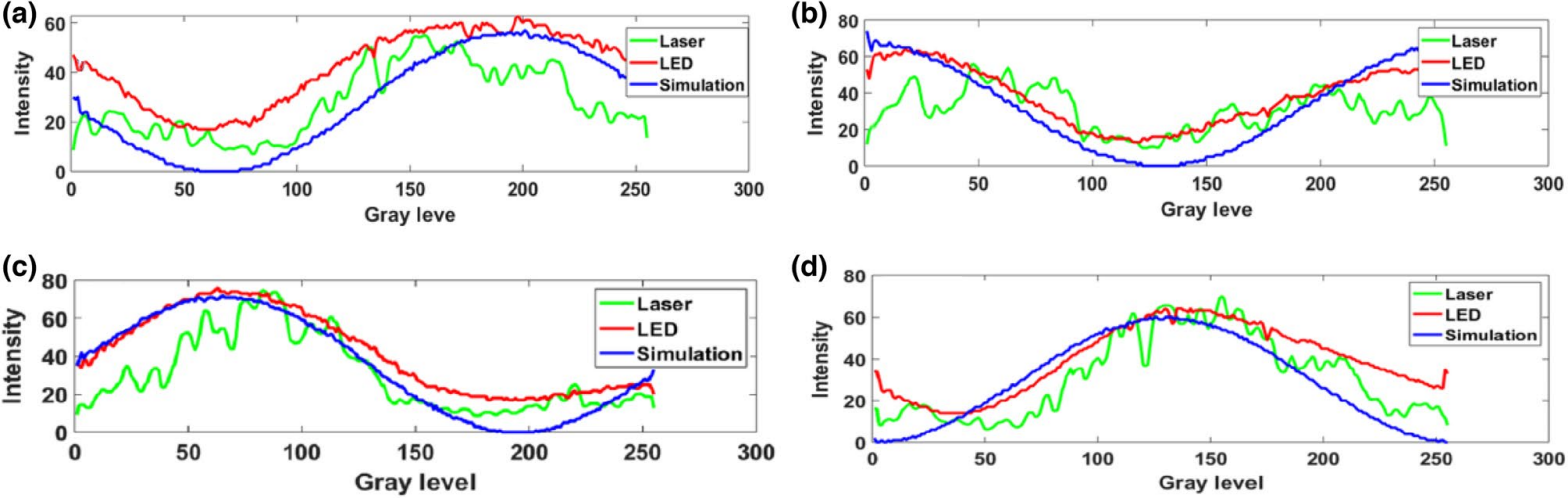
(**a**–**d**) the plot of the intensity values corresponding to different gray values for analyzer angles 0°, 45°, 90°, and 135°, respectively.

For the generation of binary polarization states, a linearly polarized light beam with a polarization angle of 45° with the *x*-axis is modulated by a phase-only SLM with an appropriate gray scale pattern. The working direction of the SLM is along the *x*-axis. A grayscale pattern having continuously increasing value from 0 to 255, as shown in Fig. 1a, is displayed into the SLM screen. The *x*-component of the light beam modulated by the SLM and the *y*-component remains as it is. After modulation, followed by a polarization basis transformation using a quarter-wave plate (QWP) aligned at 45° with the *x*-axis, the polarization state of the light beam is changed according to the grayscale pattern^42^. Thus, the amount of change in the polarization state depends upon the grayscale values. The mathematical expression of this beam can be obtained from the Jones matrix multiplication of QWP and the light beam modulated by SLM as^43^,

3 $$ \left[ {\begin{array}{*{20}c} {E_{x} (x,y)} \\ {E_{y} (x,y)} \\ \end{array} } \right] = \frac{1}{2\sqrt 2 }\left[ {\begin{array}{*{20}c} {1 - i} & { - 1 - i} \\ { - 1 - i} & {1 - i} \\ \end{array} } \right]\left[ {\begin{array}{*{20}c} {\exp \{ i\Omega (x,y)\} } \\ 1 \\ \end{array} } \right] $$

where *E*_x_ and *E*_y_ are the electric field components and *Ω*(*x*,*y*)is the POF displayed onto the SLM whose value lies within [− *π π*]. Here, the gray values from 0 to 255 are equivalent to phase values − *π* to* π*. The mathematical expression of the intensity distribution, *I* (*x*, *y*) of the encoded light beam after passing through an analyzer having transmission axis at an angle *θ* from the *x*-axis, can be expressed as^40^,

4 $$ I(\Omega ,\theta ) = \left\langle {E_{x} (\Omega )E_{x}^{*} (\Omega )} \right\rangle \cos^{2} \theta + \left\langle {E_{y} (\Omega )E_{y}^{*} (\Omega )} \right\rangle \sin^{2} \theta + \left( {\left\langle {E_{x}^{*} (\Omega )E_{y} (\Omega )} \right\rangle + \left\langle {E_{x} (\Omega )E_{y}^{*} (\Omega )} \right\rangle } \right)\sin \theta \cos \theta $$

where < … > denotes the time average. The intensity distribution, *I*(*Ω,θ*) at any point (*x*,*y*) depends on both the analyzer angle *θ* and the gray level *Ω*(*x*,*y*) corresponding to that point. For a fixed value of analyzer angle *θ*, the intensity distribution depends only on the gray level, and the spatial control of the intensity is possible by the spatial control of the gray level. The simulation and experimentally obtained intensity distributions of the light beam followed by a QWP and an analyzer after modulating the light beam by the SLM with this continually increasing grayscale pattern are shown in Fig. 2. Figure 2a–d show the simulation results of intensity distributions of the modulated light beam for analyzer angles 0°, 45°, 90°, and 135°, respectively. Figure 2e–h show the experimentally obtained intensity distributions with a coherent laser source for analyzer angles 0°, 45°, 90°, and 135°, respectively. Here, the analyzer angles are taken with respect to *x*-axis. Figure 2i–l show the experimentally obtained intensity distributions with a light emitting diode (LED) for analyzer angles 0°, 45°, 90°, and 135°, respectively. Figure 3a–d show the plot of the intensity values corresponding to different gray values for analyzer angles 0°, 45°, 90°, and 135°, respectively. The blue line corresponds to simulation result, the green line corresponds to a coherent source, and the red one corresponds to a green LED.

The obtained results suggest that all the intensity value plots corresponding to simulation, coherent source, and LED are in good agreement with each other at every analyzer angle. It is easy to find out two gray values in the case of every analyzer angle corresponding to which output intensity will have a high value and a low value. The high-intensity value point has a polarization state along the analyzer’s transmission axis and the low-intensity value point has a polarization state perpendicular to the analyzer’s transmission axis. Therefore, by appropriate selection of two grayscale values, it is possible to generate binary polarization states which are perpendicular to each other. Modulated light beam followed by an analyzer having high intensity value corresponds to the state having polarization state same as the transmission axis of analyzer while other shows low intensity. Thus, it is possible to encode any binary information into the light beam in this configuration by the appropriate choice of two grayscale values. For example, a grayscale pattern containing two gray values 70 and 200, as shown in Fig. 1b, has been displayed onto the SLM and the experimentally obtained intensity distribution at an angle 90° has been shown in Fig. 1c. This verifies the successful generation of polarization states by taking gray values 70 and 200 for the analyzer angle of 90°. From this result, it is clear that by appropriate choice of gray values, we can control the polarization states of the output light beam. Hence, binary information 0 and 1 can be encoded into the output light beam corresponding to two different polarization states which are perpendicular to each other.

### Image encryption using binary polarization states with single encryption key

An optical cryptosystem based on intensity recording has been developed. A color image of *p* × *q* pixels is used as a plaintext. The POFs corresponding to the red, green, and blue channels of plaintext have been developed with the help of MGSA, denoted as *Φ*_r_(*x*,*y*), *Φ*_g_(*x*,*y*), and *Φ*_b_(*x*,*y*), respectively. For encryption, a single POF of that plane text has been generated according to the following relation,

5 $$ \Phi_{E} (x,y) = {\text{Im}} \left[ {\log \left\{ {\exp \left( {i\Phi_{r} (x,y) + i\Phi_{g} (x,y) + i\Phi_{b} (x,y) + iK(x,y)} \right)} \right\}} \right] $$

where *K* is a random phase function used as an encryption key and ‘Im’ stands for the imaginary part of the function. To get the final encrypted data, each pixel of the POF, *Φ*_E_(*x*,*y*) is first converted into an integer number *X*^*E*^(*x*,*y*) according to Eq. (2) then convert the integer numbers into binary values. Further, the array of binary values is encoded into the light beam in the form of binary polarization states according to the above mentioned method. Thus, the final encrypted data become an array of high and low intensity values. It is not possible to get the original plaintext directly by decoding the POF *Φ*_E_(*x*,*y*) due to the presence of the random phase value distribution *K*. For correct decryption, first we have to decode the binary data from the recorded intensity of the light beam at correct analyzer angle and then convert that to the corresponding integer numbers. Now we retrieve the POF, *Φ*_E_(*x*,*y*) from that array of integer numbers according to the following relation,

6 $$ \Phi_{E} (x,y) = X^{E} (x,y)\pi /R $$

Using the above POF *Φ*_E_(*x*,*y*), POF of the plaintext corresponding to the red, green, and blue colour channels are retrieved as,

7 $$ \Phi_{r} (x,y) = {\text{Im}} \left[ {\log \left\{ {\exp \left( {i\Phi_{E} (x,y) - K_{r} } \right)} \right\}} \right] $$

8 $$ \Phi_{g} (x,y) = {\text{Im}} \left[ {\log \left\{ {\exp \left( {i\Phi_{E} (x,y) - K_{g} } \right)} \right\}} \right] $$

9 $$ \Phi_{b} (x,y) = {\text{Im}} \left[ {\log \left\{ {\exp \left( {i\Phi_{E} (x,y) - K_{b} } \right)} \right\}} \right] $$

where *K*_*r*_, *K*_*g*_, and *K*_*b*_ are the decryption keys corresponding to red, green, and blue colour channels. Mathematically, *K*_*r*_, *K*_*g*_, and *K*_*b*_ can be expressed as,

10 $$ \left. {\begin{array}{*{20}c} {K_{r} (x,y) = {\text{Im}} \left[ {\log \left\{ {\exp \left( {i\Phi_{g} (x,y) + i\Phi_{b} (x,y) + iK(x,y)} \right)} \right\}} \right]} \\ {K_{g} (x,y) = {\text{Im}} \left[ {\log \left\{ {\exp \left( {i\Phi_{r} (x,y) + i\Phi_{b} (x,y) + iK(x,y)} \right)} \right\}} \right]} \\ {K_{b} (x,y) = {\text{Im}} \left[ {\log \left\{ {\exp \left( {i\Phi_{g} (x,y) + i\Phi_{r} (x,y) + iK(x,y)} \right)} \right\}} \right]} \\ \end{array} \begin{array}{*{20}c} {} \\ \end{array} } \right\} $$

The decrypted intensity values corresponding to red channel can be given according to the following Eq. (11).

11 $$ I^{r} (x,y) = \left| {IFT\left\{ {\exp (i\Phi_{r} )} \right\}} \right|^{2} $$

Here, IFT stands for inverse Fourier transform operation. Following Eq. (11), blue and green colour channels are obtained using *Φ*_b_(*x*,*y*) and *Φ*_g_(*x*,*y*), respectively. Their combination gives the decrypted colour image.

### Enhanced security by using double encryption key

For encryption, in Eq. (5) a random phase function, *K* has been used as an encryption key. Enhancement of security has been done using one more encryption key by switching polarization state of encoded data in some randomly selected regions of the encoded beam. The encoding of binary data into the light beam has been done considering all the high-intensity values of the encoded beam as 1 and all low-intensity values as 0. But it is also possible to encode the binary data 1 as low intensity and 0 as high intensity by switching the polarization states between 1 and 0 pixels by reversing the gray values used for encoding 1 and 0.

**Figure 4:**
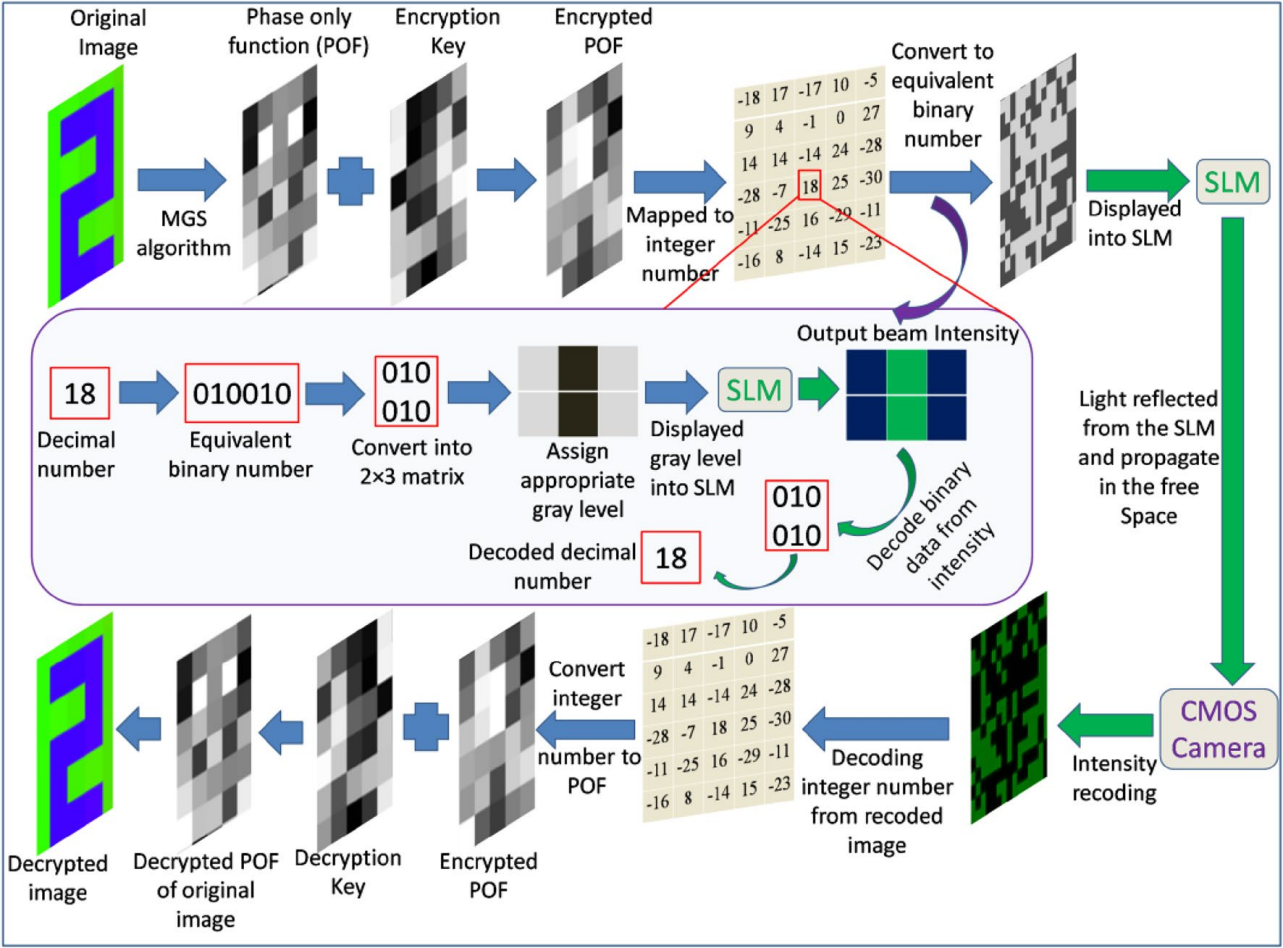
Flowchart of the encryption and decryption processes.

For employing an additional encryption key, randomly selected 50% regions of the encrypted data are encoded according to 1 as high intensity and 0 as low intensity. The remaining 50% of the encrypted data is encoded according to 1 as low intensity and 0 as high intensity. Thus, now if one knows the exact alignment of the QWP and the analyzer, but doesn’t know that 50% of randomly selected regions where polarization states are switched for encoding end up with a partially correct decoded data and it is not possible to successfully decrypt using partially correct decoded data as this scheme also involves phase retrieval algorithm. Thus, encoding by polarization switching states in some randomly selected regions in the encoded beam also act as an encryption key. The flowchart of the encryption and decryption processes has been shown in Fig. 4.

## Results and discussion

**Figure 5:**
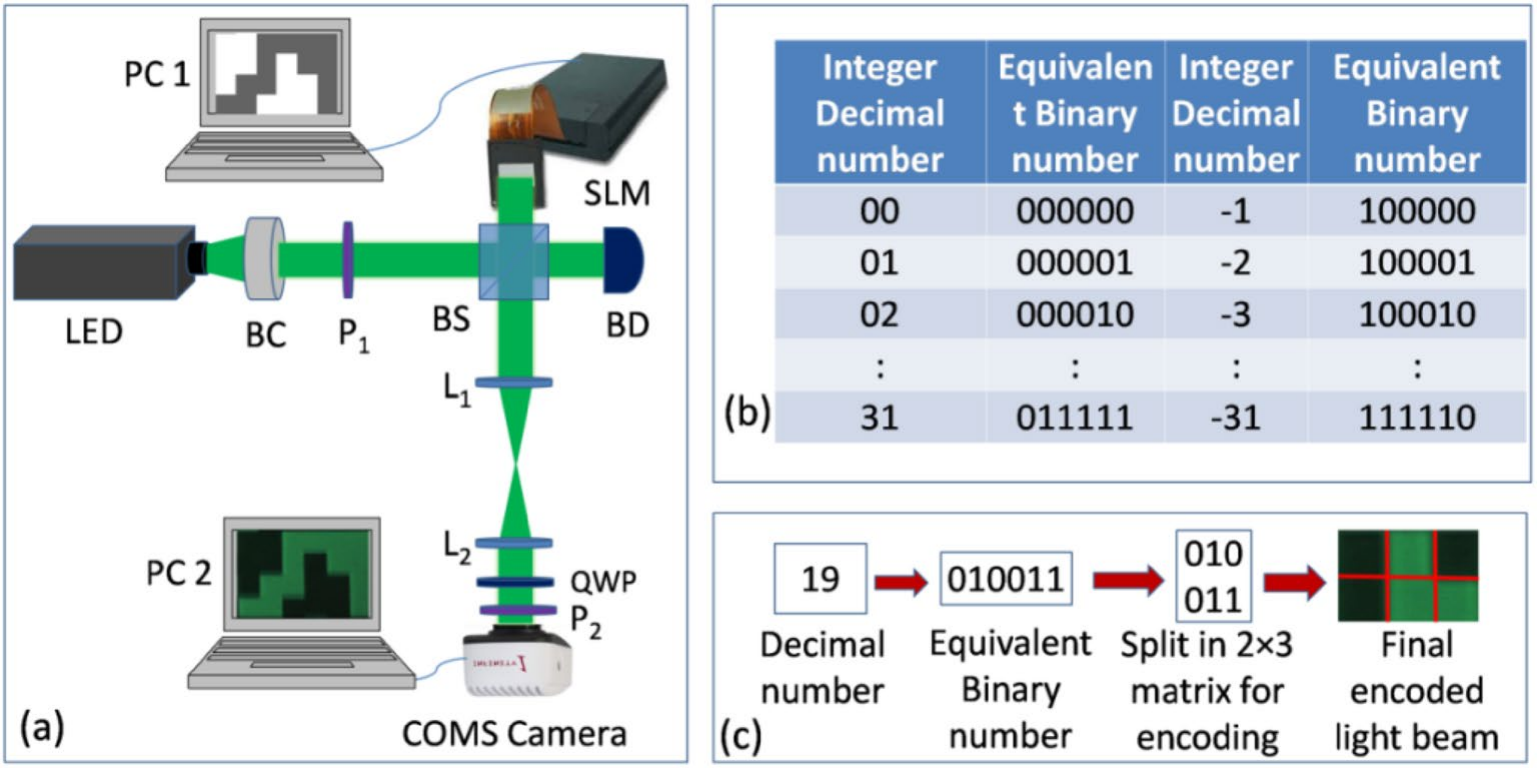
(**a**) Schematic of the experimental set-up. *BC* beam collimator, *BS* beam splitter, *BD* beam dump, *L* lens, *PC* personal computer, (**b**) list of different numbers and their equivalent binary numbers used for encoding, and (**c**) Flowchart of decimal number encoding into the light beam in the form of binary polarization states.

Figure 5a provides a schematic representation of the experimental design for the study. A reflecting type phase-only SLM (Pluto Holoeye) with a resolution of 1920 × 1080 pixels and a pixel pitch of 8.0 µm has been used to modulate the light beam according to desired binary polarization states. The *x*-axis or horizontal direction with respect to the laboratory is the direction in which SLM operates. The SLM is illuminated by an LED beam that has been collimated with a beam collimator and polarized along 45° with respect to the working direction of the SLM with a polarizer (P_1_). The complementary metal–oxide–semiconductor (CMOS) camera (Infinity, Lumenera Corp.) is then exposed to the modulated light beam for recording followed by a QWP and an analyzer (P_2_) kept at 45° and 90° with respect to *x*-axis, respectively. The camera has a 5.2 µm pixel pitch and a resolution of 1280 × 1024 pixels. In this study, the light source is a 120 µw green LED. For carrying out the experiment with a coherent light source, a collimated laser beam (DPSS laser, 100 mW; Cobolt) of wavelength 532 nm has been used. To get optimized results, a 4f imaging system has been used. The experiment uses a beam dump (BD) to block the unwanted beam. Personal computers 1 and 2 are used to control the SLM and the CMOS camera, respectively.

**Figure 6:**
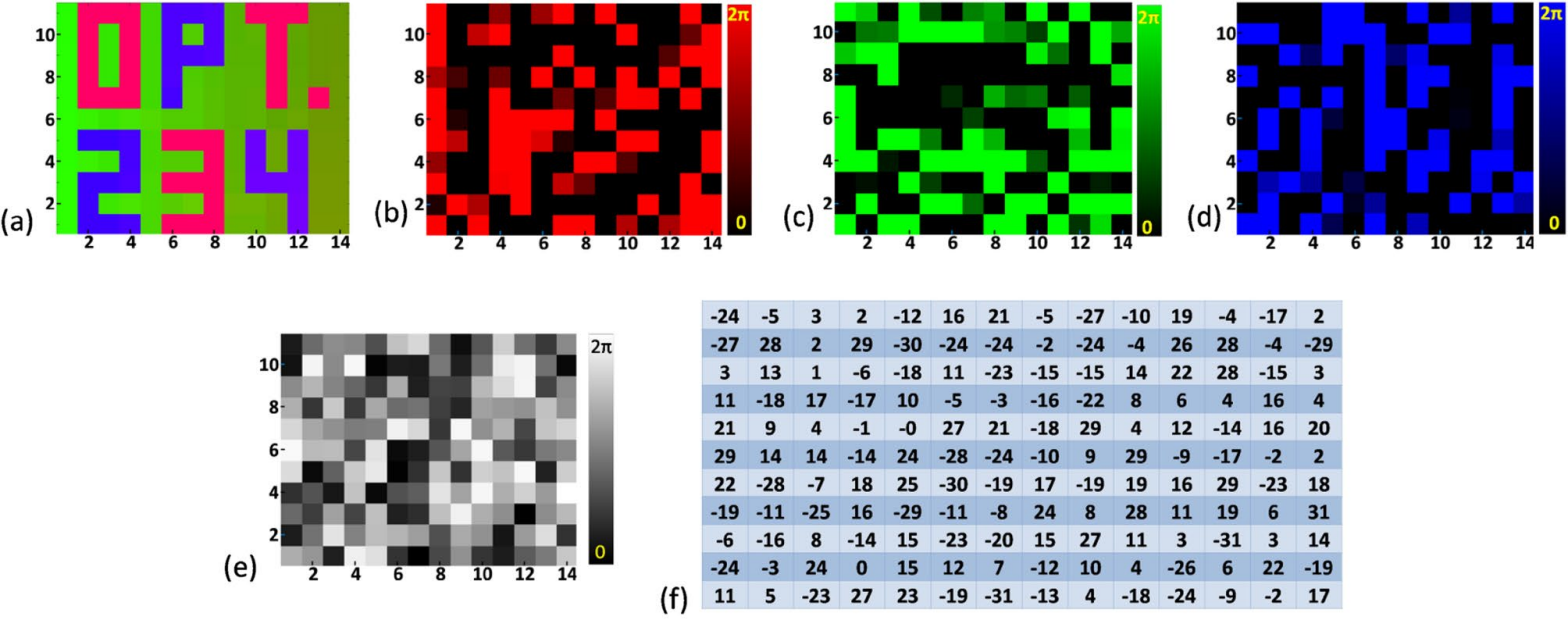
(**a**) Original image, (**b**) POF corresponding to red color channel, (**c**) POF corresponding to green color channel, (**d**) POF corresponding to blue color channel, (**e**) encrypted POF, and (**f**) integer number distribution after mapping the encrypted POF.

**Figure 7:**
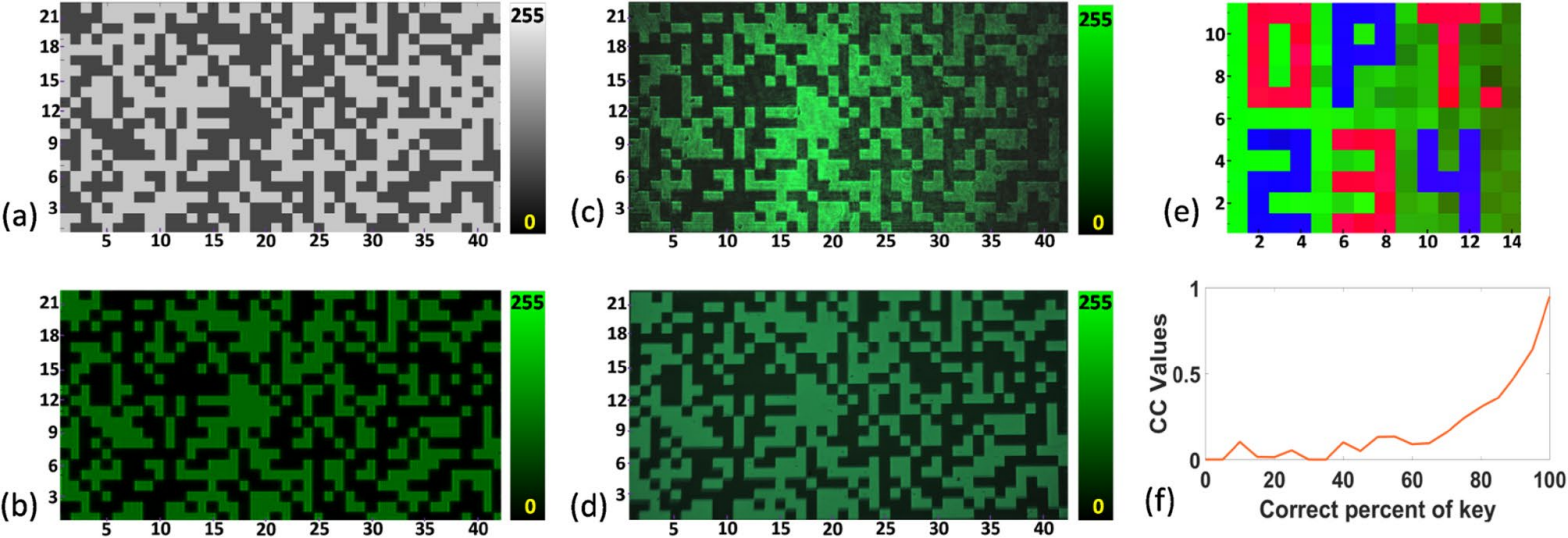
(**a**) Grayscale pattern used for encoding the array of binary data, (**b**) simulation results of the intensity distribution of the encrypted beam, (**c**,**d**) experimentally obtained intensity distribution of the intensity distribution of the encrypted beam using laser and LED, respectively, (**e**) Decrypted image, and (**f**) plot of CC values versus percentage of correct decryption key used in time of decryption.

For demonstrating the proposed method, we encrypted a color image that contains the letters and number ‘OPT.234’ of size 11 × 14 pixels, as shown in Fig. 6a. First, the POF corresponding to red, green, and blue colour channels are generated according to MGSA, as shown in Fig. 6b–d, respectively. For generating a single encrypted POF, the POFs corresponding to different colour channels are multiplexed according to Eq. (5) along with a random POF used as an encryption key. This single POF is shown in Fig. 6e. The single POF has been mapped to a generated array of integer numbers between − *R* to *R*, as shown in Fig. 6f. The loss of useful information due to discretization can be avoided by selecting a large *R*-value. Here, we take this threshold value of the integer numbers as *R* = 31. Thus, there will be 63 integer numbers between − 31 to + 31. The array of integer numbers in the Fig. 6f has been converted into equivalent binary numbers in a six digit format for encoding into the light beam. The conversion of integer number − 31 to − 1 has been done according to the flowchart shown in Fig. 5b. For utilizing maximum space of the SLM each six digit binary number is written in a 2 × 3 matrix. Figure 5c shows the flowchart of encoding decimal integer number into the light beam in the form of binary polarization states. The gray scale pattern displayed onto SLM for encoding the binary information into the light beam has been generated by replacing each 0 into gray value 200 and each 1 into gray value 70. This gray scale pattern has been shown in Fig. 7a. The gray values have been chosen arbitrarily. The intensity distribution of the light beam modulated by this gray scale pattern has been recorded by CMOS camera through a QWP and an analyzer kept at 45° and 90° with respect to *x*-axis, respectively. The simulation result of this intensity distribution has been shown in the Fig. 7b. Experimentally obtained intensity distributions using a laser light and LED are shown in Fig. 7c,d, respectively.

With the results shown in Fig. 7c,d, it is easy to identify different binary states corresponding to low and high intensity values. Analyzing the results by an inverse process, one can easily achieve the array of integer decimal number used for encoding. The decryption key has been generated according to Eq. (10). After retrieving the array of integer decimal numbers from the experimentally obtained intensity distribution and using the decryption key it is easy to decrypt different colour channels according to Eq. (11). The combination of the red, green, and blue colour channels results the final decrypted image, as shown in Fig. 7e.

**Figure 8:**
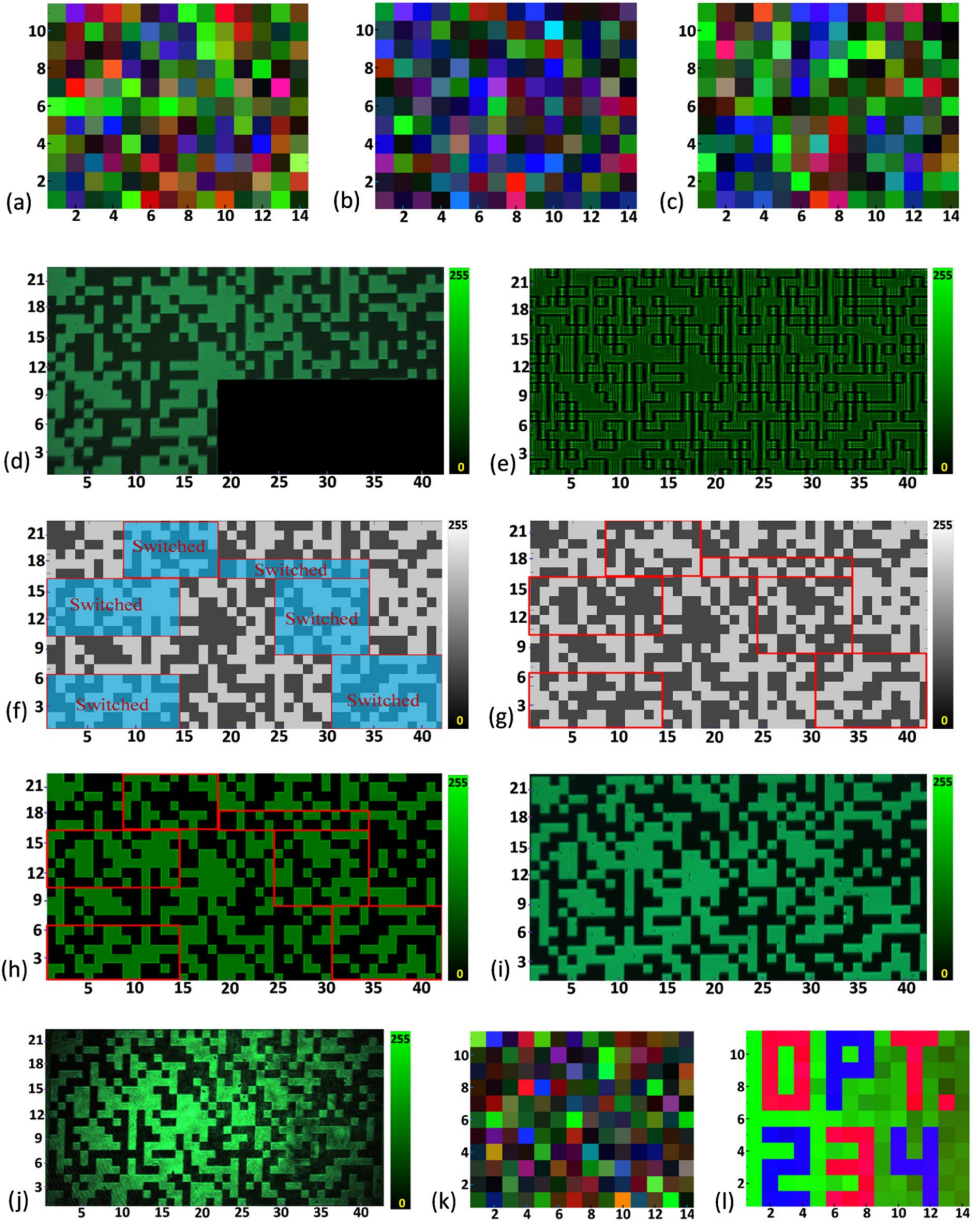
(**a**) Decrypted image using 25% wrong decryption key, (**b**) decrypted image in case of misalignment of the array of integer numbers, (**c**,**d**) decrypted image with 25% loss of encrypted beam and experimentally obtainted intensity distribution of the encrypted beam, (**e**) intensity distribution recorded without using QWP and analyzer, (**f**) polarization switched region of the gray scale pattern, (**g**) gray scale pattern after switched gray values, (**h**) simulated intensity distribution of the encoded beam after polarization switching, (**i**,**j**) experimentally obtained results of intensity distribution of the encoded beam after polarization switching using LED and laser source, respectively, (**k**) decrypted image obtained without polarization switching, and (**l**) decrypted image obtained after polarization switching.

The correlation coefficient (CC) value is a measure of the agreement between two data sets. The CC value between two data sets as 1 means the data sets completely match each other and its value 0 indicates no match. In our case, the CC value between the original image and the decrypted image 0.97 indicates good decryption and the CC value between the original image and the encrypted image 0.014 indicates good encryption. Figure 7f shows the plot of CC values between the original and the decrypted images with respect to the correct percentage of keys used in decryption. In this plot, it is seen that to get CC value more than 0.5 requires more than 90% of correct key. This indicates the effectiveness of the encryption system. Figure 8a shows the decrypted result using 25% wrong decryption key which indicates that it is not possible to extract original information using a partially correct decryption key. Figure 8b shows the decrypted result in case of misalignment in the array of integer numbers, which confirms that misalignment of the array of integer causes loss of information.

We further investigate the situation if some part of the encrypted text is missing or lost. Figure 8c shows the decrypted information corresponding to 25% loss of encrypted data and Fig. 8d shows the intensity distribution of the encrypted beam with 25% loss of data, which means even partial loss of data in decryption key becomes a barrier to successful decryption. Figure 8e shows the simulated intensity distribution of the encrypted beam without the QWP and analyzer. Here, it is not possible to identify the binary states. Thus, the information of the particular angle of the QWP and the analyzer angle are also important for correct decryption.

For employing the second encryption key, around 50% regions of the encrypted data is encoded with opposite polarization states. Those regions are randomly selected and shown by the red boxes in Fig. 8f. Figure 8g shows the gray scale pattern after switching the gray levels for those regions. Figure 8h shows the simulated intensity distribution of the encoded beam after switching the polarization states of those particular regions. The region inside the red box shown in Fig. 8h gives a low-intensity value corresponding to the binary information 1 and a high-intensity value corresponding to the binary information 0, while the other regions of the encoded beam return high-intensity values corresponding to the binary information 1 and low-intensity values corresponding to the binary information 0. Therefore, if anyone doesn’t know the particular polarization state switched regions will end up with wrong encrypted information. Figure 8i,j show the experimentally obtained intensity distributions of the encoded beam after switching the polarization states of those particular regions using LED and laser as a light source, respectively. Figure 8k shows the decrypted image without knowing any information about those particular polarization states switched regions but applying the correct first decryption key. Figure 8l shows the decrypted image considering the correct first decryption key and correct information about those particular polarization states switched regions. This indicates that the polarization switch of some random regions of the encrypted beam can act as an additional encryption key and enhanced the security.

**Figure 9:**
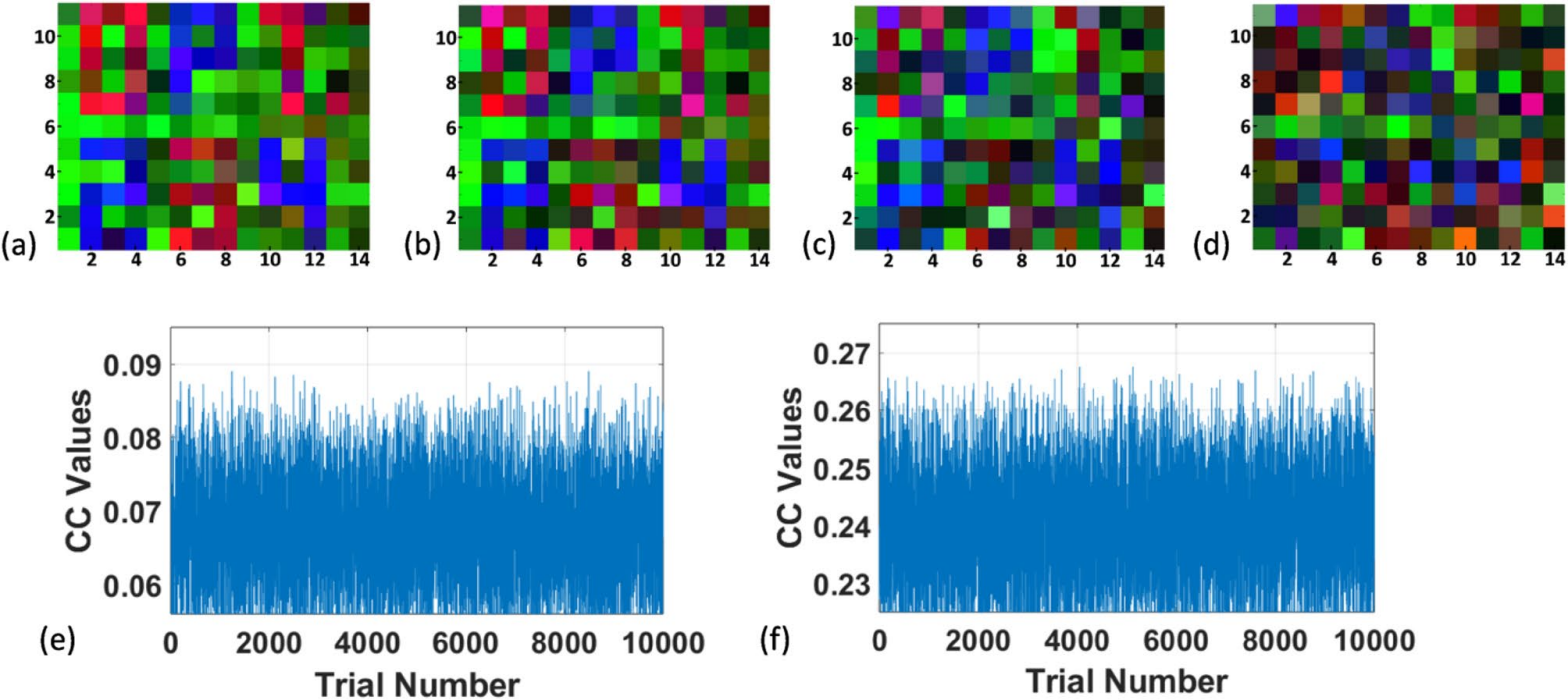
(**a**–**d**) decrypted images with 95%, 90%, 85%, 80% of correct key pixels, respectively, (**e**) plot of CC values between plaintext and decrypted images as a function of the number of decryption trials with random keys, and (**f**) plot of CC values between plaintext and decrypted images as a function of the number of decryption trials with the decryption keys contain 75% correct and 25% random information each time.

The results of the key sensitivity test are illustrated in Fig. 9a–d. During decryption, certain elements of the key were replaced with random values. In Fig. 9a, the decrypted image is partially readable which has been achieved with 95% correct key. Figure 9b–d display decrypted images using 90%, 85%, and 80% of the correct key pixels, respectively. The results of Fig. 9d corresponding to decryption with 80% correct key pixels indicate all most unsuccessful decryption. These findings demonstrate that a partially correct key might decrypt some pixels of the plaintext but it fails to provide fully readable information. Further, simulation study has been carried out where several decryption trials have been performed with random keys with different possible combination. The plot of CC values between decrypted and original images with the number of trials is shown in Fig. 9e, which indicates that even with 10,000 trials, the maximum and mean CC value remains 0.09 and 0.07, respectively. Similarly Fig. 9f shows the plot of CC values between decrypted and original images with the number of trials where the decryption key contains 75% correct and 25% random information each time. This result indicates that even with 10,000 trials, the maximum and mean CC value remains 0.268 and 0.24, respectively. The analysis shows the efficiency of the proposed scheme to encrypt color images while maintaining the decryption quality securely.

## Conclusion

In conclusion, we propose a single-shot intensity recording-based encryption scheme using binary polarization states with the help of a single SLM. Here, the POF of the original image encoded in the form of a binary data enhances the quality of the decoded image compared to encoding the POF directly into the light beam using a digital phase modulator. It uses a straight forward and non-interferometric set-up and is possible to perform using low-cost partially coherent light like LED. It is not possible to decrypt the whole information if a small portion of the encoded text or decryption key is manipulated as it involves modified GS algorithm. Enhancement of security has been ensured by switching polarization states in some randomly selected regions of the encrypted beam and used it as an additional encryption key. The study paves the way for communicating optical data securely in free-space.

## Data Availability

Data underlying the results presented in this paper are not publicly available at this time but may be obtained from the authors upon reasonable request.
